# Pulmonary arterial hypertension in children with congenital heart disease: a deeper look into the role of endothelial progenitor cells and circulating endothelial cells to assess disease severity

**DOI:** 10.3389/fped.2023.1200395

**Published:** 2023-07-06

**Authors:** Juan Calderón-Colmenero, Felipe Massó, Héctor González-Pacheco, Julio Sandoval, Carlos Guerrero, Jorge Cervantes-Salazar, José A. García-Montes, Araceli Paéz, Gabriela I. Pereira-López, Carlos Zabal-Cerdeira, Juan Pablo Sandoval

**Affiliations:** ^1^ Department of Pediatric Cardiology, Instituto Nacional de Cardiologia Ignacio Chavez, Mexico City, Mexico; ^2^ Department of Molecular Biology, Instituto Nacional de Cardiologia Ignacio Chavez, Mexico City, Mexico; ^3^ Coronary Unit, Instituto Nacional de Cardiologia Ignacio Chavez, Mexico City, Mexico; ^4^ Department of Cardiopulmonary Medicine, Instituto Nacional de Cardiologia Ignacio Chavez, Mexico City, Mexico; ^5^ Department of Cardiovascular Surgery in Congenital Heart Disease, Instituto Nacional de Cardiologia Ignacio Chavez, Mexico City, Mexico

**Keywords:** circulating endothelial cells, endothelial progenitor cells, pulmonary arterial hypertension, congenital heart disease, down syndrome

## Abstract

Endothelial progenitor cells and circulating endothelial cells have been proposed as useful markers of severity and disease progression in certain vascular diseases, including pulmonary arterial hypertension. Our study focused on evaluating the levels of circulating endothelial progenitor cells and circulating endothelial cells in patients with congenital left-to-right shunts and pulmonary hypertension undergoing definitive repair. Endothelial progenitor cells (identified by simultaneous co-expression of CD45dim, CD34 + and KDR2 + surface antibodies) and circulating endothelial cells (identified by simultaneous co-expression of inherent antibodies CD45-, CD31+, CD146 + and CD105+) were prospectively measured in seventy-four children (including children with Down syndrome), median age six years (2.75–10), with clinically significant left-to-right shunts undergoing transcatheter or surgical repair and compared to thirty healthy controls. Endothelial progenitor cells and, particularly, circulating endothelial cells were significantly higher in children with heart disease and pulmonary arterial hypertension when compared to controls. Endothelial progenitor cells showed significant correlation with pulmonary vascular resistance index when measured both systemically (*r* = 0.259; *p* = 0.026) and in the superior vena cava (*r* = 0.302; *p* = 0.009). Children with Down syndrome showed a stronger correlation between systemic cellularity and pulmonary vascular resistance index (*r* = 0.829; *p* = 0.002). Endothelial progenitor cells were reduced along their transit through the lung, whereas circulating endothelial cells did not suffer any modification across the pulmonary circulation. In children with yet to be repaired left-to-right shunts, endothelial progenitor cells and circulating endothelial cell counts are increased compared to healthy subjects.

## 1. Introduction

Endothelial progenitor cells (EPC) and circulating endothelial cells (CEC) have been recently proposed as useful markers of severity and disease progression in different forms of pulmonary arterial hypertension (PAH) (1, 2), including patients with PAH associated to congenital heart disease (PAH-CHD) (3–9). Limited information exists regarding the balance between EPC and CEC in PAH-CHD when compared to healthy subjects. Furthermore, their actual significance regarding PAH severity before definitive repair has been established remains yet to be determined. We sought to prospectively investigate the role of these cells as biomarkers of disease severity in children with PAH associated to the presence of left-to-right shunts undergoing transcatheter or surgical correction.

## 2. Methods

### 2.1. Study population

We included children aged 2 to 18 years with clinically significant left-to-right shunts with varying degrees of hemodynamic impact. Clinical and demographic characteristics, along with a complete physical and echocardiographic assessment were recorded. All patients underwent a diagnostic right and left heart study (cardiac catheterization) for complete baseline hemodynamic evaluation and operability assessment (10–12). Acute vasoreactivity testing and/or pulmonary wedge angiography were performed in cases of near-systemic or systemic pulmonary hypertension. Blood samples were purposefully drawn from each patient from the pulmonary artery, the superior vena cava and either the femoral artery or pulmonary vein (i.e., systemic) for subsequent EPC and CEC analysis. Amenable defects (i.e., atrial septal defect and patent ductus arteriosus) underwent uneventful transcatheter device occlusion; the remainder underwent subsequent surgical repair. Control subjects (*n* = 30) consisted in children that were sent to our institution based on suspected heart disease that was ruled out after proper evaluation. Peripheral vein blood samples were drawn from controls to determine EPC and CEC count. This prospective study was supported by the National Council of Science and Technology (CONACYT); under the Sectorial Fund for Health Research and Social Security (FOSSIS No. 234296). Our study was approved by the Internal Review Board (approval no. 15–917) in compliance of all the appropriate steps taken to protect the rights and welfare of participating subjects in the research. Parental informed consent (and patient assent when appropriate) where provided in accordance with the principles outlined in the Declaration of Helsinki.

### 2.2. Identification, characterization and quantification of endothelial progenitor cells and circulating endothelial cells

**Figure 1:**
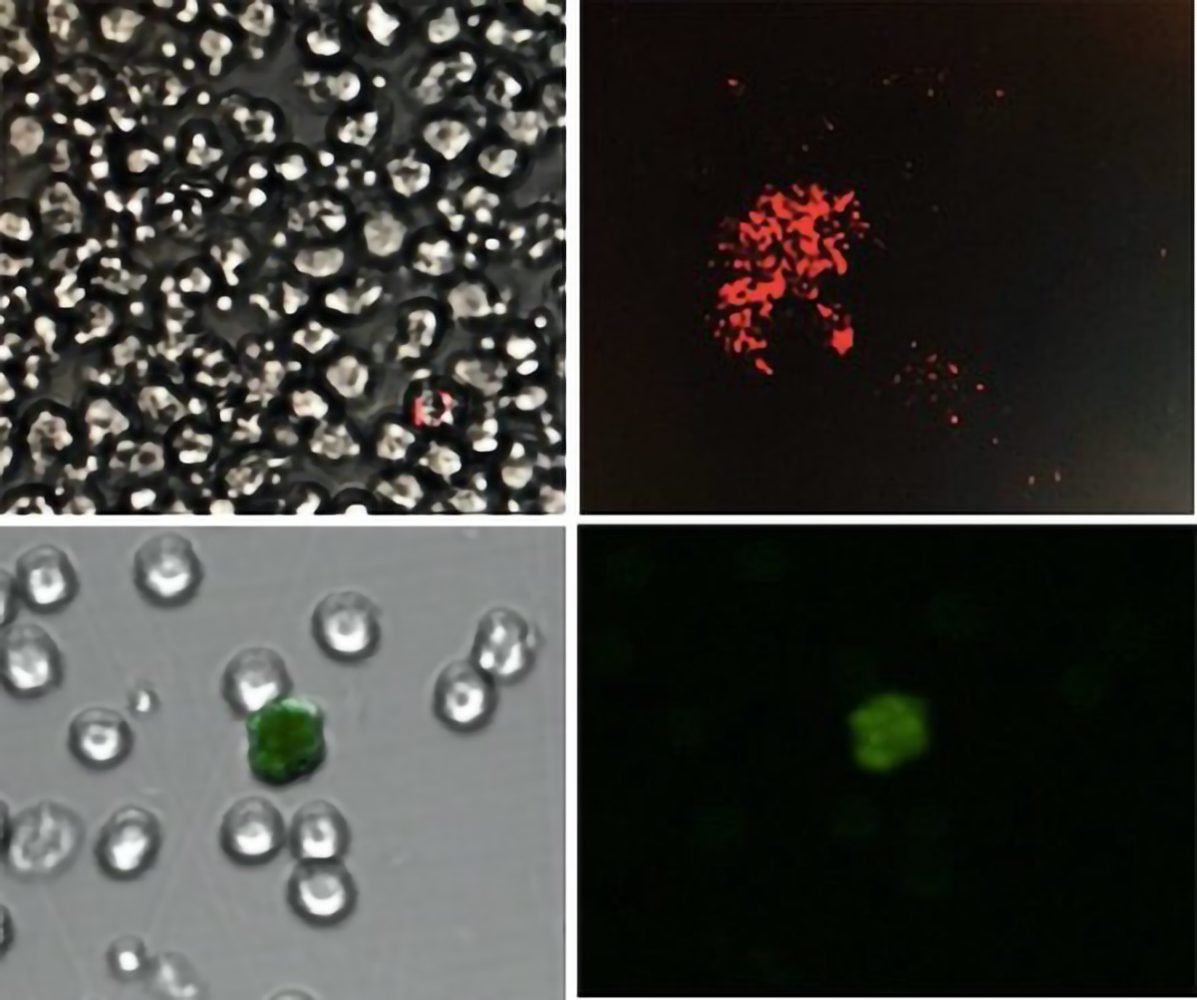
Upper panel: single endothelial progenitor cell with double marker (left). Positive CD34 + surface antibody marker (red) (right). All EPC were identified by simultaneous co-expression of CD45dim, CD34 + and KDR2 + . Lower Panel: Mononuclear cells surrounding single circulating endothelial cell marked in green with CD146 (FITC) surface antibody under a clear (left) and dark (right) field. CEC were identified by simultaneous co-expression of inherent antibodies including CD45-, CD31+, CD146 + and CD105 + .

Immunostaining flow cytometry was used for cellular characterization and quantification (13–18). EPC were characterized according to the modified ISHAGE (International Society of Hematotherapy and graft engineering) protocol. Peripheral Bone Marrow Mononuclear Cells (PBMNC) were isolated from whole blood sample by a Hystopaque density 1.077 gradient (Sigma Chemical St. Louis MO. USA). Plot sized (FSC-H) vs. granularity (SSC-H) were obtained to identify low granularity medium-sized cells (lymphocyte area) (R1). Following one-hour incubation, an aliquot of approximately 1 × 106PBMNC with 4 microliters of the following antibodies: CD45-PercP, CD34-PE and KDR2-APC (Biolegend, San Diego CA, US) was obtained. Dot plot SSC-H vs. CD34-PE (FL2-H) was performed to determine CD34+ (R2). An additional dot plot SSC-H vs. CD45-PercP (FL3-H) was acquired to identify CD45-CD45dim and CD45bright cells, ultimately creating a window to locate CD45dim cells (R3). EPC were identified provided all events were fulfilled at the 3 gates: R1 (medium size and low granularity) R2 (CD34 +) and R3 (CD45dim). The latter were then analysed in a dot plot FL2-H (CD34+) vs. FL4-H (KDR2+) and considered all positive cells those located in the upper right quadrant (i.e., positive for all surface markers) (Figure 1, upper panel). A total of 250.000 events were counted for each sample. Quantified cells are expressed as the number of EPC per million events. Circulating endothelial cells were characterized following a similar protocol. An aliquot of approximately 1 × 106PBMNC was incubated for one hour with 4 microliters of the following antibodies: CD45-PercP, CD105-PE, CD31-APC and CD146 FITC (Biolegend, San Diego CA, US). A dot plot SSC-H (granularity) vs. FL3-H (CD45-PercP) was performed to accurately locate region CD45- (R1). Since both leukocytes and mature endothelial cells are CD31 +, but solely CEC are CD45-, subsequent dot plot FL3-H (CD45-PercP) vs. FL4H (CD31-APC) was carried out with positively marked cells located in the upper left quadrant (negative for CD45 but positive for CD31) (R2). Finally, from this window (R2 region), a dot plot FL1-H (CD146-FITC) and FL2-H (CD105-PE) was obtained to determine all positive cells in the right upper quadrant and labelled as CEC (CD45-, CD31+, CD146+, CD105+) (Figure 1, lower panel). A total of 250.000 events were counted for each sample. Quantified cells are expressed as the number of CEC per million events.

Detailed analysis was performed comparing the number of EPC and CEC according to type of shunt and site of sampling, with particular focus on correlation between the type and number of cells and hemodynamic impact. Furthermore, we compared groups according to the presence (or absence) of PAH as per its most recent definition of a mean pulmonary artery pressure ≥20 mmHg and pulmonary vascular disease (defined by a pulmonary vascular resistance index (PVRi) ≥than 3 Wood units). Lastly, comparison between patients with DS and non-syndromic children was thoroughly analysed.

### 2.3. Statistical analysis

All categorical data were summarized as frequencies and percentages. All continuous variables were tested and confirmed to have a non-normal distribution as determined by the Shapiro-Wilks test. Continuous variables were reported as medians and 25th and 75th percentiles (interquartile ranges, IQRs). Statistical differences across groups were assessed, either using the chi-square or Fisher's exact tests in the case of categorical variables or the Kruskal–Wallis or Mann–Whitney *U* tests, as appropriate, for continuous variables. The strengths of correlation between cellularity and pulmonary hemodynamic were assessed using Spearman's rank correlations. Results were reported using two-tailed significance; statistical significance was set at *P* ≤ 0.05. All analyses were performed using SPSS version 17.0 statistical software (Chicago, IL, USA).

## 3. Results

Seventy-four children [median age 6 years (2.75–10)] with clinically significant left-to-right shunts were prospectively studied between December 2016 and December 2018. Defects included patent ductus arteriosus (*n* = 39); ventricular septal defect (*n* = 13); atrial septal defect (*n* = 18), atrioventricular septal defect (*n* = 2), aortopulmonary window and truncus arteriosus (*n* = 1 each). Eleven (15%) patients were children with Down syndrome. Demographic and clinical characteristics along with total EPC and CEC determination of patients vs. healthy controls (*n* = 30) are shown in Table 1. EPC and CEC counts were significantly higher in children with PAH-CHD when compared to controls. Hemodynamic left-to-right shunt impact and cellularity behavior influenced by mean arterial pulmonary pressure are shown in Table 2. There exists a predominance of post-tricuspid defects in the total cohort of patients and children with higher pulmonary pressures. As expected, pulmonary hypertension was significantly higher in all children with DS. Interestingly, other than lower determinations of CEC from the superior vena cava in patients with higher pulmonary artery pressures, we found no other relevant differences regarding cellularity between both groups. Comparably, pulmonary vascular disease, defined as pulmonary vascular resistance index (PVRi)> 3 WU was found in approximately 20% of children with CHD. Table 3 shows patients with higher PVRi were predominantly male, all having post-tricuspid shunts and children with DS tended to be more prevalent in this group. Similarly, EPC and CEC counts were not significantly different when comparing these groups according to PVRi. A separate analysis comparing children with DS and non-syndromic children is shown in Table 4. The former attest to higher systolic and mean pulmonary pressures but also to increased EPC within the pulmonary artery and significantly lower concentrations of CEC at the superior vena cava.

**Table 1:** Baseline demographic and clinical characteristics of patients vs. controls.

Variable, Median (IQR) or *n* (%)	Controls (*n* = 30)	Patients (*n* = 74)	*p* value
Age, years	13 (11–16)	6 (2.75–10)	0.001
Gender, female	15 (50)	43 (58)	0.451
BSA, m^2^	1.56 (1.31–1.71)	0.74 (0.52–1.0)	0.001
Left-to-right shunt			
PDA	–	39 (53)	
ASD	–	18 (24)	
VSD	–	13 (18)	
AVCD	–	2 (2.7)	
APW		1 (1.4)	
TA	–	1 (1.4)	
Down syndrome	–	11 (14.9)	
EPC, cells/10^6^ peripheral blood	24 (19–32)	34 (20–56)	0.011
CEC, cells/10^6^ peripheral blood	0 (0–4)	52 (24–81)	0.001

BSA, body surface area; PDA, patent ductus arteriosus, ASD, atrial septal defect; VSD, ventricular septal defect; AVSD, atrioventricular septal defect; APW, aorto-pulmonary window; TA, truncus arteriosus; EPC, endothelial progenitor cells; CEC, circulating endothelial cells.

**Table 2:** Clinical, hemodynamic, and cellular analysis in children with left-to-right shunts, with and without pulmonary arterial hypertension.

Variable, median (IQR) or *n* (%)	Total group (*n* = 74)	PAP < 20 mmHg (*n* = 23)	PAP ≥ 20 mmHg (*n* = 51)	*p* value
Age, years	6 (2.7–10)	8 (6–12)	4 (2–7)	0.001
Gender, Female	43 (58)	8 (35)	35 (69)	0.006
BSA, m^2^		0.96 (0.82–1.3)	0.57 (0.51–0.82)	0.001
Diagnosis, *n* (%)				
PDA	39 (53)	7 (30)	32 (63)	0.001
VSD	13 (18)	1 (4)	12 (23)	
AVSD	2 (3)	1 (4)	1 (2)	
APW	1 (1.4)	0 (0)	1 (2)	
ASD	18 (24)	14 (60.9)	4 (8)	
TA	1 (1.4)	0 (0)	1 (2)	
Down syndrome, *n* (%)	11 (15)	0 (0)	11 (22)	0.016
Systolic PAP, mmHg	43.5 (30–56)	28 (22–30)	55 (40–60)	0.001
Mean PAP, mmHg	28 (18–35)	15 (15–17)	30 (27–39)	0.001
PVRi, WU	2.15 (1.5–2.72)	2.0 (1.6–2.2)	2.2 (1.5–2.9)	0.332
PVRi/SVRi	0.16 (0.10–0.22)	0.12 (0.08–0.15)	0.17 (0.14–0.24)	0.003
Qp/Qs	3.5 (2.4–5.1)	2.3 (1.7–3.3)	3.9 (3.2–5.7)	0.001
EPC cells/10^6^				
SVC	34 (20–56)	32 (20–44)	36 (24–64)	0.200
PA	58 (32–85)	48 (32–84)	64 (32–88)	0.761
Systemic	40 (20–49)	40 (20–56)	40 (20–48)	0.652
CEC cells/10^6^				
SVC	52 (24–81)	68 (52–100)	36 (24–72)	0.010
PA	56 (28–87)	48 (44–104)	60 (28–84)	0.739
Systemic	44 (28–90)	56 (40–104)	44 (20–88)	0.054

PAP, pulmonary artery pressure; PVRi, pulmonary vascular resistance index; WU, wood inits; PVRi/SVRi, pulmonary vascular resistance index/systemic vascular resistance index ratio; Qp: Qs, pulmonary to systemic flow ratio; EPC, endothelial progenitor cells; SVC, superior vena cava; PA, pulmonary artery; Systemic (blood sample obtained from femoral artery or pulmonary vein); CEC, circulating endothelial cells. Rest of abbreviations as Table 1.

**Table 3:** Clinical, hemodynamic, and cellular analysis in children with left-to-right shunts with and without pulmonary vascular disease.

Variable, median (IQR) or *n* (%)	Total group (*n* = 74)	PVRI < 3WU (*n* = 61)	PVRI ≥ 3WU (*n* = 13)	*p* value
Age, years	6 (2.75–10)	4 (2.5–8)	8 (3–13)	0.080
Gender, Female	43 (58)	39 (64)	4 (31)	0.030
Diagnosis, *n* (%)				
PDA	39 (53)	34 (56)	5 (38)	0.003
VSD	13 (18)	7 (11)	6 (46)	
AVSD	2 (3)	1 (2)	0 (0)	
APW	1 (1.4)	0 (0)	1 (8)	
ASD	18 (24)	18 (29)	0 (0)	
TA	1 (1.4)	0 (0)	1 (8)	
Down syndrome, *n* (%)	11 (15)	7 (11)	4 (31)	0.076
Systolic PAP, mmHg	43 (30–56)	40 (30–55)	60 (37–67)	0.066
Mean PAP, mmHg	28 (18–35)	27 (17–32)	36 (21–44)	0.011
PVRi, WU	2.15 (1.5–2.72)	2.0 (1.4–2.3)	3.9 (3.2–4.75)	0.000
PVRi/SVRi	0.16 (0.1–0.22)	0.15 (0.1–0.18)	0.29 (0.2–0.3)	0.000
Qp/Qs	3.5 (2.4–5.07)	3.5 (2.4–5.4)	3.5 (1.74–3.7)	0.345
EPC cells/10^6^				
SVC	34 (20–56)	32 (20–54)	44 (26–82)	0.159
PA	58 (32–85)	60 (34–86)	44 (30–78)	0.504
Systemic	40 (20–49)	36 (18–48)	44 (24–74)	0.119
CEC cells/10^6^				
SVC	52 (24–81)	52 (24–88)	52 (28–70)	0.904
PA	56 (28–87)	56 (28–98)	56 (30–68)	0.599
Systemic	44 (28–90)	48 (28–92)	44 (22–92)	0.792

Abbreviations as in Tables 1, 2.

**Table 4:** Clinical, hemodynamic, and cellular analysis in non-syndromic vs. children with Down syndrome with left-to-right shunts.

Variable, median (IQR) or *n* (%)	Total group (*n* = 74)	DS (*n* = 11)	non-syndromic (*n* = 63)	*p* value
Age, years	6 (2.75–10)	6 (3–7)	6 (2–10)	0.860
Gender, Female	43 (58)	6 (54)	37 (59)	0.795
Diagnosis, *n* (%)				
PDA	39 (53)	4 (36)	35 (56)	0.078
VSD	13 (18)	5 (45)	8 (13)	
AVSD	2 (3)	1 (9)	1 (2)	
APW	1 (1.4)	0 (0)	1 (2)	
ASD	18 (24)	1 (9)	17 (27)	
TA	1 (1.4)	0 (0)	1 (2)	
Systolic PAP, mmHg	43 (30–56)	60 (50–60)	40 (30–55)	0.011
Mean PAP, mmHg	28 (18–35)	35 (28–40)	24 (17–33)	0.015
PVRi, WU	2.15 (1.5–2.72)	2.4 (1.7–3.7)	2 (1.5–2.6)	0.176
PVRi/SVRi	0.16 (0.10–0.22)	0.25 (.19-.3)	0.15 (.10-.21)	0.059
Qp/Qs	3.5 (2.4–5.1)	3.7 (3.4–6.4)	3.3 (2.3–4.9)	0.138
EPC cells/10^6^				
SVC	34 (20–56)	48 (28–56)	32 (20–56)	0.290
PA	58 (32–85)	72 (68–88)	44 (28–84)	0.030
Systemic	40 (20–49)	44 (32–68)	36 (16–48)	0.065
CEC cells/10^6^				
SVC	52 (24–81)	32 (20–44)	60 (28–92)	0.020
PA	56 (28–87)	60 (36–84)	48 (28–96)	0.354
Systemic	44 (28–90)	32 (16–96)	48 (28–88)	0.442

DS, down syndrome. Rest of abbreviations as listed in Tables 1, 2.

### 3.1. Correlation between cellularity and pulmonary hemodynamic

**Figure 2:**
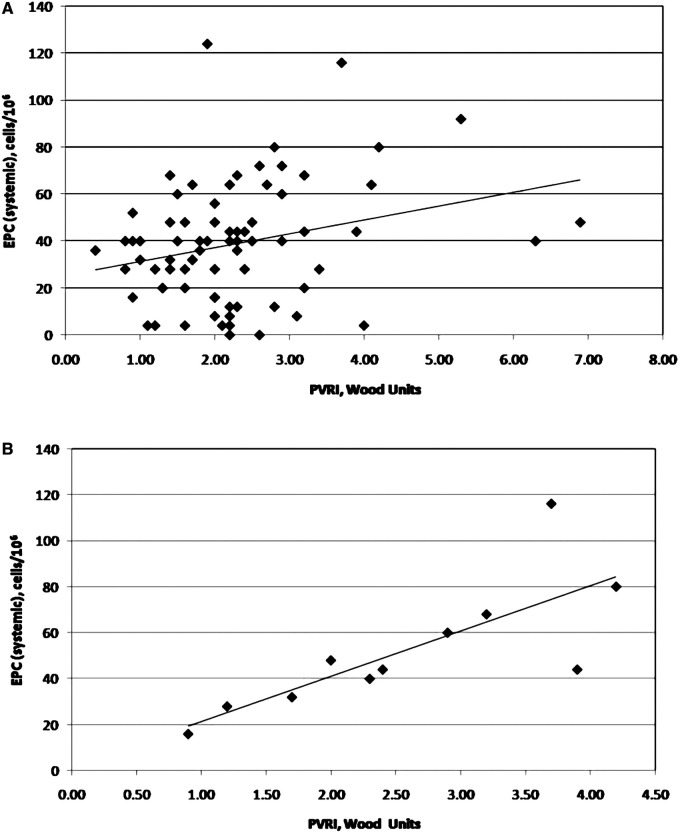
Correlation between endothelial progenitor cells and pulmonary vascular resistance index (as a reflection of potential vascular disease) in patients with congenital left-to-right shunts. Figure 2A shows the nature of this correlation in the total patient population (*n* = 74; *r* = 0.259; *p* = 0.026); Figure 2B shows the same correlation in patients with Down syndrome (*n* = 11; *r* = 0.829; *p* = 0.002).

In the whole group, age (*r* = 0.383; *p* = 0.001) and BSA (*r* = 0.398; *p* = 0.001) correlated with EPC when measured in the systemic circulation. Also, EPC were shown to have a significant correlation with PVRi when measured both systemically (*r* = 0.259; *p* = 0.026) (Figure 2A) and in the superior vena cava (*r* = 0.302; *p* = 0.009). No significant correlation was found between EPC and pulmonary artery pressures (either systolic or median) or the pulmonary to systemic flow ratio (Qp:Qs). In children with DS, correlations between cellularity and hemodynamic variables behaved differently. There was a strong correlation between EPC at systemic level and PVRi (*r* = 0.829; *p* = 0.002) (Figure 2B) and CEC tended to correlate with PVRi when measured in the superior vena cava (*r* = 0.526; *p* = 0.096). In similar fashion to the total cohort, no significant correlations were found between children with DS regarding EPC or CEC and pulmonary artery pressure.

### 3.2. The lung circulation as a determinant of systemic cellularity

Obtaining blood samples at the pulmonary artery and either the femoral artery or the pulmonary vein (i.e., systemic) allowed us to explore the potential role of the pulmonary circulation to determine the final cellularity by performing simple subtraction of systemic—pulmonary artery cell counts. The results of this analysis are shown in Table 5. Overall, EPC are reduced along their journey through the lung in the total cohort including patients with and without pulmonary hypertension. Circulating endothelial cells, on the other hand, do not seem to suffer any significant modification across the lung including patients with PAH or pulmonary vascular disease.

**Table 5:** EPC and CEC journey across the lung.

Median, (IQR)	Pulmonary artery	Systemic	Gradient	*p*-value
Total group (*n* = 74)				
EPCs, cells/10^6^	58 (32–85)	40 (20–49)	20 (−5–40)	0.001
CECs, cells/10^6^	56 (26–87)	44 (28–90)	4 (−22–41)	0.347
Endothelial Progenitor Cells				
PAP <20 (*n* = 23)	48 (32–84)	40 (20–56)	22 (−8–40)	0.026
PAP ≥20 (*n* = 51)	64 (32–88)	40 (20–48)	20 (0–38)	0.001
PVRi <3 UW (*n* = 61)	60 (34–86)	36 (18–48)	24 (0–44)	0.000
PVRi ≥3 UW (*n* = 13)	44 (30–78)	44 (24–74)	8 (−14–24)	0.462
Circulating Endothelial Cells				
PAP <20 (*n* = 23)	48 (44–104)	56 (40–104)	4 (−32–44)	0.583
PAP ≥20 (*n* = 51)	60 (28–84)	44 (20–88)	4 (−22–40)	0.320
PVRi <3 UW (*n* = 61)	56 (28–98)	48 (28–92)	4 (−23–44)	0.420
PVRi ≥3 UW (*n* = 13)	56 (30–68)	44 (22–92)	4 (−24–28)	0.637

Abbreviations, as in Table 2.

## 4. Discussion

It has been established that measurement of systemic EPC and CEC may provide an estimate of individual vascular competence that results from the delicate balance between injury and repair and may, in turn, yield valuable prognostic clues in certain vascular diseases including several forms of pulmonary hypertension (13, 16). It has also been widely accepted that while CEC are biomarkers of damage (with higher determinations predicting poor outcome) (16, 19), EPC are biomarkers of repair with therapeutic potential (with low levels predicting worse outcome) (16, 20, 21). Our study focused to evaluate the levels of circulating EPC and CEC in patients with congenital left-to-right shunts and pulmonary hypertension undergoing definitive repair. To this end we undertook a careful characterization of EPC and CEC using a variety of surface markers at different sample sites during baseline diagnostic (pre-surgical) or interventional catheterization. The main findings of our study can be summarized and prioritized as follows: (1) EPC and CEC counts are higher than normal in children with unrepaired left-to-right shunts (2) The number of EPC correlated with age, BSA, and baseline PVRi (3) The severity of pulmonary arterial hypertension or pulmonary vascular disease did not seem to influence cellularity in the group as a whole and (4) Patients with DS not only had more severe pulmonary arterial hypertension and higher EPC count, but the relationship between pulmonary vascular disease and cellularity was stronger.

### 4.1. The role of EPC and CEC in children with pulmonary arterial hypertension associated to congenital heart disease

**Figure 3:**
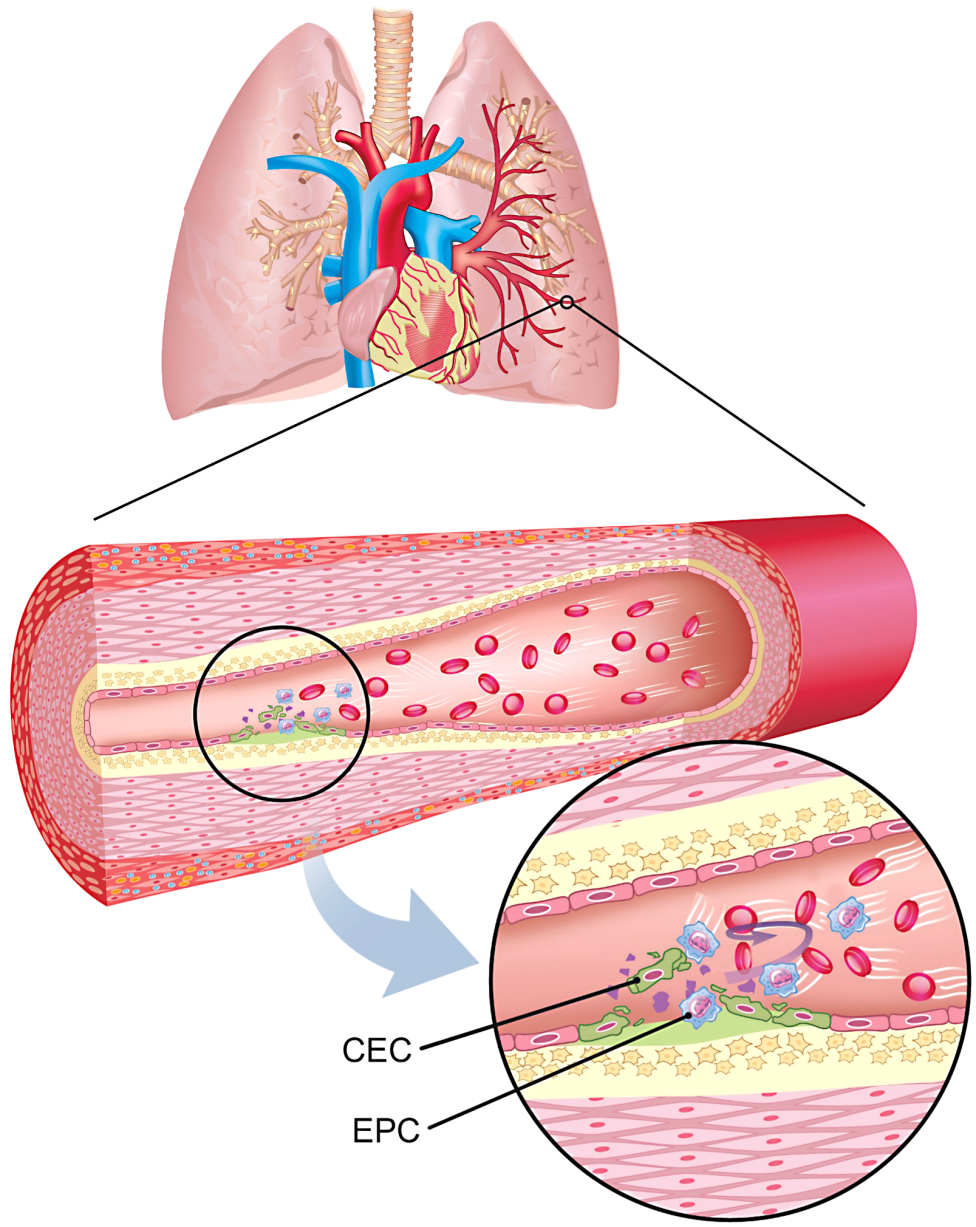
Schematic representation of the delicate and ongoing balance between vessel injury and repair between endothelial progenitor cells (EPC) and circulating endothelial cells (CEC) in the pulmonary endothelium in the setting of children with left-to-right shunts and pulmonary arterial hypertension.

Previous studies have emphasized the role of EPC and CEC in the setting of idiopathic pulmonary arterial hypertension and PAH-CHD. 1–9 Smajda et al. 4, 5 and Levy et al. 8 (within the same group of investigators) demonstrated that CEC counts are increased and may well be defined as exclusive markers of irreversible PAH in patients with PAH-CHD (as defined by the persistence of pulmonary hypertension at least six months after repair) or patients with definitive idiopathic PAH. In contrast, they found CEC counts in patients with reversible PAH (normalized pulmonary artery pressures after repair) were low and not any different from controls. Furthermore, they highlighted relevant implications regarding PAH therapy, particularly reduction of CEC in patients with irreversible forms of PAH when started on PAH-specific medications and thus useful to monitor response to treatment. 7, 8 Also, a recent case-control study by Xiaofel- Li et al, 9 found a direct and significant correlation between the mean pulmonary artery pressure and the pulmonary to systemic flow ratio (Qp:Qs) and CEC levels in patients with PAH-CHD. Similarly, the role of EPC in the setting of idiopathic PAH and PAH-CHD (including Eisenmenger syndrome) has also been explored. Diller et al. 3 have shown that EPC counts are reduced in these patients when compared to healthy controls. Altogether, these studies strongly suggest that in the setting of advanced stages of pulmonary vascular disease, EPC are reduced impairing the intrinsic reparative capability of the pulmonary endothelium, whereas increased CEC are proper reflection of established vascular damage. Interestingly and unlike Xiaofel Li et al, 9 we did not find any significant correlation between CEC and important hemodynamic variables (i.e., systolic or mean pulmonary artery pressure and PVRi). We believe the latter, can be partially explained because irreversible vascular damage in our patients has not yet been established. If anything, there was a weak but statistically significant correlation between EPC counts and PVRi in the whole group suggesting an elevated number of EPC in patients (in need of repair) with higher PVRi. However, it is important to stress out that EPC and CEC counts were not different when we analyzed them separately in groups with and without pulmonary arterial hypertension or in groups with and without pulmonary vascular disease. Our findings regarding elevated counts of both EPC and CEC rather suggest that the delicate process of injury and repair is ongoing (13, 16, 17). This is further supported by the gradient found in the cellular count while on transit between the pulmonary and the systemic circulation we have demonstrated in this study. This important finding suggests that many of these cells, particularly EPC are retained on their pass through the lung (Figure 3). We speculate that these “retained cells” might actively participate in the process of healing or even in the process of proliferation and further remodelling (22–24). Currently, mechanisms of how bone marrow-derived or resident cell lines are attracted-to and/or retained in the lung are being investigated (25–28). Patients in our cohort will certainly have long-term follow up and we will hopefully be able to further address and support this concept.

### 4.2. EPC and CEC in the setting of children with down syndrome and left-to-right shunts

Prevalence of congenital heart defects in children with DS vary from 44% to 58%, with a preponderance of persistent left-to-right shunts (single or in combination) that represent approximately 85% of all cardiac anomalies found in this population (29). It has well been established, children with DS have an increased risk for developing pulmonary hypertension, naturally due to the presence of these high pulmonary blood-flow driven lesions but also due to additional non-cardiac factors including chronic upper airway obstruction and abnormal pulmonary vascular growth (30, 31). In particular, EPC and CEC determination in children with DS and pulmonary hypertension remains somewhat controversial. Both Smadja et al. 4 and Xiaofel-Li et al. 9 excluded patients with DS from their studies. Their decision was based on potential bias regarding accurate cellular determination according to what they suggest involves an abnormal production of different hematopoietic lines (including EPC) within the bone marrow niches of children with DS. This argument is supported by an interesting study that showed diminished EPC production (approximately 40%) in children with DS (interestingly, without heart disease or PH) when compared to healthy non-syndromic subjects (32). Furthermore, Diller et al. 3 analyzed EPC in 41 patients (13 of them with DS) with established Eisenmenger syndrome, 55 patients with idiopathic PAH and 47 healthy subjects. Their findings highlight reduced EPC counts in patients with Eisenmenger syndrome when compared to healthy controls and even lower in patients with DS. Our findings are in keeping showing higher pulmonary artery pressures in children with DS but are dramatically different regarding elevated EPC counts in this subgroup of patients. As aforementioned, a significant correlation between EPC and pulmonary vascular resistance index was also found in our study, reflecting perhaps a greater degree of endothelial damage and consequently the need of early repair in this population. It is fair to assume however, that DS patients in our cohort were still in a so-called reversible state of pulmonary hypertension without an established Eisenmenger physiology that may somehow explain the differences between our results and those found in Diller's et al. 3.

## 5. Summary and conclusions

In children with yet to be repaired left-to-right shunts, EPC and CEC counts are increased when compared to healthy subjects albeit cellular counts at this stage cannot clearly establish the degree of severity of elevated pulmonary artery pressure and/or pulmonary vascular resistance, except perhaps for children with DS. The nature of our findings regarding elevated counts of EPC and CEC in children with PAH-CHD rather suggests that the delicate process of endothelial injury and repair is continuous and intricately related. To this end, ongoing pulmonary vascular damage might be eventually suppressed once the shunt is repaired and the stimuli arising from shear stress imposed by increased pulmonary blood flow and consequently pressure is no longer present.

One of the limitations of our study is that the control group is older than the group of patients studied (median age 13 vs. 6 years old), one variable that can confound comparisons between groups, but this can be explained by the early onset of symptoms in patients with congenital heart diseases with left to right shunt compared with the control group which consisted in healthy children that were sent to our institution based on suspected heart disease that was ruled out after proper evaluation.

The strength of this study and the aim of it is that there is a group of patients in which we don't know the evolution after the congenital defect has been repaired despite all the evaluation before the procedure that supports clinical decision. That is why we suggest that determination of endothelial progenitor cells and circulating endothelial cells can be an additional tool in decision-making that can help predict the long-term results of patients with congenital heart disease and left-to-right shunt.

## Data Availability

The raw data supporting the conclusions of this article will be made available by the authors, without undue reservation.
